# Single-Molecule Dynamics Reveals Cooperative Binding-Folding in Protein Recognition

**DOI:** 10.1371/journal.pcbi.0020078

**Published:** 2006-07-07

**Authors:** Jin Wang, Qiang Lu, H. Peter Lu

**Affiliations:** 1 State Key Laboratory of Electroanalytical Chemistry, Changchun Institute of Applied Chemistry, Chinese Academy of Sciences, Changchun, Jilin, People's Republic of China; 2 Department of Chemistry and Department of Physics, State University of New York at Stony Brook, Stony Brook, New York, United States of America; 3 Pacific Northwest National Laboratory, Fundamental Science Division, MSIN K8-88, Richland, Washington, United States of America; Cornell University, United States of America

## Abstract

The study of associations between two biomolecules is the key to understanding molecular function and recognition. Molecular function is often thought to be determined by underlying structures. Here, combining a single-molecule study of protein binding with an energy-landscape–inspired microscopic model, we found strong evidence that biomolecular recognition is determined by flexibilities in addition to structures. Our model is based on coarse-grained molecular dynamics on the residue level with the energy function biased toward the native binding structure (the Go model). With our model, the underlying free-energy landscape of the binding can be explored. There are two distinct conformational states at the free-energy minimum, one with partial folding of CBD itself and significant interface binding of CBD to Cdc42, and the other with native folding of CBD itself and native interface binding of CBD to Cdc42. This shows that the binding process proceeds with a significant interface binding of CBD with Cdc42 first, without a complete folding of CBD itself, and that binding and folding are then coupled to reach the native binding state. The single-molecule experimental finding of dynamic fluctuations among the loosely and closely bound conformational states can be identified with the theoretical, calculated free-energy minimum and explained quantitatively in the model as a result of binding associated with large conformational changes. The theoretical predictions identified certain key residues for binding that were consistent with mutational experiments. The combined study identified fundamental mechanisms and provided insights about designing and further exploring biomolecular recognition with large conformational changes.

## Synopsis

Biomolecular function (e.g., binding) is often thought to be determined by the underlying molecular structure. There are more and more findings that molecular binding sometimes involves large conformational changes in various stages of cell function. Addressing this issue will answer the critical questions about how molecular function is determined by conformational flexibility and dynamics in addition to structure. Combining a single-molecule fluorescence study of flexible protein binding with an energy-landscape–inspired microscopic molecular dynamics model, the authors found strong evidence that biomolecular recognition is determined by flexibility and large conformational changes in addition to structure. The single-molecule study shows conformational fluctuations of the protein complex that involve bound and loosely bound states, which can be quantitatively explained in the authors' model as a result of cooperative binding. Theoretical predictions about the key residues are consistent with mutational experiments. Identifying the key residues for binding provides a structural basis for designing drugs that will target those critical residues.

## Introduction

The study of associations between two biomolecules (e.g., proteins, RNA, or DNA) is essential in understanding molecular recognition and function. A standard paradigm, which has been successfully applied for many enzyme proteins, is that of a molecular function such as binding determined by the structure of the molecule. The lock-and-key mechanism of binding assumes that two biomolecules maintain rigid structures during association [1]. The induced-fit mechanism [2] suggests that biomolecules can adjust their conformations to a limited extent during the association. In nature, however, the binding between two biomolecules is often accompanied by large conformational changes in various stages of the cell function, including cell proliferation, differentiation, and death. It has been estimated [3–6] that up to 30% of proteins, when isolated, are in their unfolded or partially disordered forms. Since the native binding complex is usually well structured, given that the isolated form of the proteins before binding is not well structured, the binding process from nonnative states toward the native state (structure) involves large conformational changes (from unstructured or unfolding states to well-structured or native folding states during binding). The flexibility or disordered forms of the proteins in the cells can be targeted for rapid turnover, thus providing an additional lever of control. Here, flexibility rather than rigidity is crucial for binding as well as for biological function. However, the flexible binding processes are not yet well understood. Addressing this issue will answer the critical questions about how molecular function is determined by conformational flexibility and dynamics in addition to structure.

Cell signaling is at the core of most biological functions and often involves dynamic interactions among proteins. Protein–protein interactions induce conformational changes that initiate chain reactions, which, in turn, lead to cellular responses. Characterization of such protein interactions is critical to the understanding of the regulatory mechanisms that control cellular functions. To study protein interactions in cell signaling, ensemble measurements, which yield information only on averaged properties, are inadequate. The crucial early events of cell signaling often involve only a few molecules and then are magnified along the signaling pathways. Furthermore, for intrinsically heterogeneous systems such as protein complexes, protein interaction dynamics possesses both spatial and temporal inhomogeneities [7–10], which result in inhomogeneous rates among protein complexes (static inhomogeneity) and rate fluctuations during the time course of protein–protein interaction (dynamic inhomogeneity).

Single-molecule spectroscopy is powerful in deciphering such complex dynamics [11–14], making it ideal for studying conformational dynamics and localization of proteins under physiological conditions. In a recent study, the interactions significant to recognition of an intracellular signaling protein, Cdc42, with its downstream effector protein, the CBD fragment of Wiskott-Aldrich syndrome protein (WASP) [15–18] labeled with a solvatochromic dye, were probed using single-molecule fluorescence spectroscopy [19–21]. Cdc42 belongs to the Rho family of small guanosine 5-triphosphate (GTP)-binding proteins (GTPases) that act as molecular switches in signaling pathways to regulate diverse cellular responses [15–18]. Only when it is bound to GTP does Cdc42 assume an active conformation that enables it to bind and activate a series of effector proteins via direct protein–protein noncovalent interactions [15–18]. Previous nuclear magnetic resonance and X-ray crystallographic analysis and studies of binding structures provided a knowledge base to facilitate our interpretation of the single-molecule data [15–18].

The single-molecule study of Cdc42 and the CBD fragment of WASP (PDB name: 1cee) shows significant static and dynamic conformational fluctuations for the binding from the bound and loosely bound states, which suggests that biomolecular recognition involves a highly flexible protein tertiary structure, and the binding domains undergo dramatic conformational changes from disordered to ordered states upon complex formation [19–21]. It is possible that structural transitions of flexible conformational domains are common in biomolecular recognition, which can be identified by measuring single-molecule conformational fluctuation dynamics.

For flexible binding, an important question is how the huge number of configurations could fall to the unique native state. The most natural and simple way of resolving this so-called Levinthal paradox [22] was originally proposed for protein folding: the underlying energy landscape is funneled against the roughness or traps to guarantee both thermodynamic stability and specificity [4–6,23–26]. This would also lead to faster kinetics. The funnel is believed to originate from evolutionary selection so that unstable and nonfunctional complexes with bumpy underlying landscapes would be evolutionarily unfavorable. Only the complexes with funneled landscapes against traps could survive, being relatively stable and performing specific biological functions.

In a perfect funneled binding landscape, the structural heterogeneity of the intermediate states, such as transition state ensembles and partially folded and binding states, seems mostly determined by geometrical constraints (or constraints of native structure), reflecting more a balance between the entropy of forming spatial contacts and the uniform or overall binding stabilization energy rather than the heterogeneity of the energetics. In other words, information on the intermediate structure ensembles can be reasonably inferred when the native structure of the binding complex is known. The Go model [27,28] was proposed to emphasize the importance of native structure in determining the interaction energies. The Go model assumes that the interaction energies among residues that are spatially close in the native structure are attractive and uniform in strength without energetic heterogeneity. The Go model has been proven to be consistent with many experimental findings, including two-state and three-state folding thermodynamic stability and kinetics [28–36], the role of contact order and topology in folding [37,38], and φ value analysis for identifying key residues for folding [28–31,34].

It is worthwhile to point out that although the Go model assumes a smooth underlying energy landscape, the complex multi-exponential kinetics can emerge in three or more states in the free-energy profile. Free energy is composed of both energy and entropy. Since kinetics are determined by the free energy, the resulting complex kinetics must be from the entropic contribution to free energy. The entropic contribution for folding comes from constraining the polypeptide chain from a free to a native structure. Obviously, the native structure is not uniform. Thus, the heterogeneity in the structure provides the source of heterogeneity of the entropy and, therefore, the ruggedness of the free energy in the Go model, even when the energetics are uniform and smooth. The heterogeneity in the native structure provides the source of the so-called topological frustration—a particular configuration might not be favored by all other configurations. In other words, certain topological constraints might be in conflict with others so that they can be hard to satisfy everywhere.

It was expected that the Go model would also provide useful information about the global topology of the underlying binding energy landscape [4–6]. As mentioned, from many previous studies, the Go model results are quite consistent with the experiments. This implies that the global topology of the native structure is very important in determining the underlying folding mechanisms. This might be a general principle revealed by the Go model. It is likely that the binding mechanism is also largely determined by the global topology in general [4–6]. Thus, for our system of Cdc42 interacting with CBD, we will explore the dynamics of binding with the Go model and compare it with the experiments.

## Results and Discussion

### Characterizing the Cdc42-CBD Complex

We previously used a dye-labeled fragment of WASP (denoted CBD, for the Cdc42 binding domain of WASP) to track Cdc42 activity and protein–protein interactions in the binding complex [19–21]. CBD, which is a 13-kDa WASP fragment (residues 201–320), contains the CRIB (Cdc42/RAC interactive binding) motif (residues 238–251), an N-terminal portion (residues 201–237), and a C-terminal segment (residues 252–320), with dye labeling at residue 271 via a cysteine mutation. This biosensor was designed for live-cell imaging based upon a domain-dye approach that is advantageous for studying unlabeled proteins in vivo, and a novel solvatochromic dye, I-SO (indolenine-benzothiophen3-one-1,1-dioxide), whose fluorescence properties are sensitive to changes in the local environment [19]. For the single-molecule experiments, Cdc42-CBD protein complexes at nanomolar concentrations were embedded in agarose gel (0.5%) and sandwiched between two cleaned cover glasses.

Collecting fluorescence emission images and photon-stamping time trajectories, and applying auto-correlation function analysis [19–21], we were able to probe and analyze the conformational fluctuation dynamics at the protein–protein interaction interface of individual Cdc42-CBD complexes. On the basis of a series of control experiments [19–21], we have attributed the dynamics of the single-molecule Cdc42-CBD conformational fluctuations to both bound and loosely bound states of the protein complex (Figure 1) [19–21]. Our experimental results suggest that the loosely bound states were presumably a subset of conformations that deviate from the bound equilibrium states without rupturing the interactions, so the overall protein complex is still associated partially. It was concluded that the bound and loosely bound conformational states correspond to different degrees of distribution of the solvent-accessible surface and that the loosely bound states are more solvent-accessible [19–21]. The spectroscopic characterization of the bound and loosely bound states shows them to be analogues of states of Cdc42-CBD and CBD alone measured in controlled ensemble-averaged experiments [19–21]. Although it is still difficult to identify exactly how many conformational states contribute to the inhomogeneous distribution, we have postulated that there are at least two subgroups of states associated with conformational fluctuations, as illustrated in Figure 2 [19–21].

**Figure 1:**
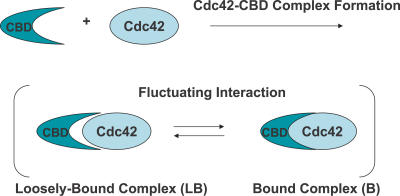
An Illustration of Two State Cdc42-CBD Binding The loosely bound state was presumably a subset of conformations that deviated from the bound equilibrium states, probed by the dye, without disrupting the subnanometer long-range interactions, so the overall protein complex was still associated.

**Figure 2:**
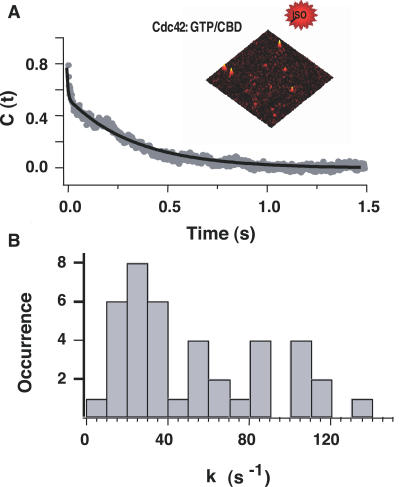
Single-Molecule Fluorescence and Spectroscopy Studies of Cdc42-CBD Protein–Protein Interaction Dynamics (A) Second-order auto-correlation function, C(t), calculated from a fluorescence intensity trajectory I(t) of a single Cdc42-CBD complex. Solid curve is a bi-exponential fit. (Inset) A single-molecule fluorescence raster-scanning image of GTP-loaded Cdc42 in complex with dye-labeled CBD biosensor. (B) The occurrence histogram of the single-complex conformational fluctuation rate for 60 individual protein complexes [19].

### Characterizing the Underlying Binding Energy Landscape of the Cdc42-CBD Complex

To further identify the bound and loosely bound states or subgroups in Cdc42-CBD protein–protein interaction dynamics (Figure 1), we conducted residue-level Go model molecular dynamics (MD) simulations. These simulations explored the underlying binding energy landscape of the Cdc42-CBD complex. In order to describe the flexible binding, at least three reaction coordinates are required: *Q _I_* as the fraction of native spatial tertiary contacts representing the degrees of freedom of the interface binding; *Q _f_*_1_ as the fraction of native spatial tertiary contacts representing the degree of freedom of folding or flexible conformational changes of the Cdc42; and *Q _f_*_2_ as the fraction of native spatial tertiary contacts representing the degrees of freedom of folding or flexible conformational changes of the CBD complex (Figure 3A).

**Figure 3:**
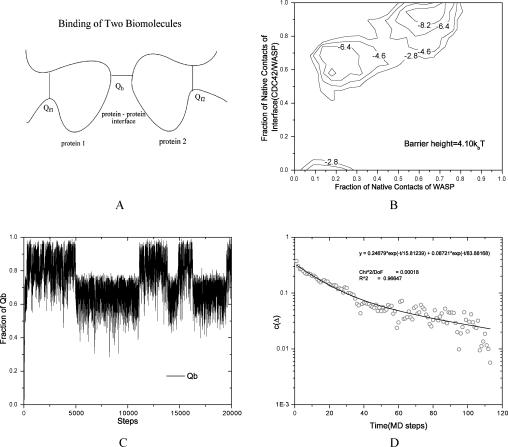
Illustration of Flexible Binding-Folding through Two-Dimensional Free-Energy Landscape, Trajectory and Time Correlation (A) Flexible binding-folding. (B) Two-dimensional free-energy contour plots of the interface degree of freedom between CBD portion of WASP and Cdc42, and conformation/folding degree of freedom for CBD. (C) Typical trajectory of fraction of native contacts of binding interface between CBD portion of WASP and Cdc42. (D) Time correlation function of fraction of native interface contacts.

The two-dimensional free-energy contour plot provided a more complete picture of the binding process. In the wide temperature range in which we performed the simulations, the Cdc42 was stable in its native state, and we considered only folding and binding of CBD and the interface between CBD and Cdc42. Figure 3B shows the free-energy contour at the binding-folding transition temperature as a function of the fraction of native contacts of the binding interface (vertical axis, mimicking the degrees of progression of native binding ) and the fraction of native contacts of the folding (horizontal axis, mimicking the degrees of progression of native folding ) of the CBD. If the binding-folding process is noncooperative, then the gradient of the free-energy profile would lead, first, to moving along the axis of the folding degree of freedom and, second, to following the axis of the binding degree of freedom. This usually results in at least three stable free-energy states: the unbound-unfolded state, the intermediate nearly folded but not bound state, and the native binding state. This leads often to multi-exponential kinetics. If the binding-folding process is cooperative, then the gradient of the free-energy profile would drive the binding-folding path to move through the middle of the two-dimensional free-energy contour (on a nearly diagonal path) toward the native state. This process usually results in only two free-energy stable states: the unbound-unfolded state and the native binding state. Therefore, the kinetics are often found to be exponential. In extreme cases, the binding-folding process will proceed largely along the binding axis before subsequently completing the folding. Our case is close to this one where the protein (CBD) is unstructured or unstable before binding. Only upon very significant binding can the stable folding form and the native stable binding complex of Cdc42-CBD be formed. Folding needs the help of binding. In this extreme case, at least three states (the unbound-unfolded state, the partially bound, largely unfolded state, and the native binding state) coexist. The multi-exponential kinetics can thus emerge.

Notice that a quantitative measure of the cooperative binding-folding can thus be defined from a binding path in a two-dimensional binding-folding map. We can set the horizontal axis as folding and the vertical axis as binding. The origin is the unbound-unfolded state. If the binding path moves through the lower right corner to reach the final native state at the upper right corner (native state) in the map, then the binding-folding process is less cooperative. If the binding path moves through the diagonal line toward the upper right corner (native state), then the binding-folding process is cooperative. If the binding path proceeds toward the upper right corner of the map from the upper left corner of the map, then binding is prerequisite for stable folding. Therefore, binding causes folding, and the binding-folding process is cooperative.

The underlying free-energy landscape of the binding clearly shows the distinct conformational states as partially folded (*Q_f_*_2_ = 0.2; 0.2 here means 20% native) with no binding (*Q_I_* = 0), partially folded (*Q_f_*_2_ = 0.3) with partial native binding (*Q_I_* = 0.7), and mostly folded (*Q_f_*_2_ = 0.7) with native binding (*Q_I_* = 0.9). This shows that folding and binding do not proceed independently (individually) but are intimately coupled. It is more likely that the whole binding process progresses first as the partial folding of CBD to a very limited amount (only 20%, mostly through local folding), then as significant interface binding (70%, without much further folding), and finally as binding and folding cooperatively to the native state. When considered in the light of our theoretical calculated free-energy minimum, the past experimental findings [19–21] of the dynamic fluctuations between the loosely bound and closely bound conformational states correspond to the cooperative binding-folding process, disorder-to-order transition of CBD upon binding. Cooperative binding-folding coupled with inherent hydrophobic interactions leads to the formation of the two states (loosely bound and bound) and provides the basis and micro-origin of conformational changes seen in single-molecule experiments [19–21]. These cooperative interactions among residues are mediated by water molecules. They can be mimicked by three-body interactions among residues instead of simple two-body interactions [39,40,41]. Taking into account the multibody nature of hydrophobic interactions (three-body interactions are considered in this study), the resulting free-energy barrier separating these two basins is significant (4.1 kT).

The two conformational basins near the native state were found to be quite broad, implying that there are many conformational substates in each conformational basin (Figure 3B and 3C). Thus, there appear to be many competing processes with slightly different or distributed barrier heights for both interbasin and intrabasin transitions. The dynamics are complex, with both the interbasin transitions and the intrabasin transitions occurring simultaneously. The kinetic process is thus likely to be multi-exponential (see Figure 3C for trajectories in time and Figure 3D for correlation function in time), which is consistent with the kinetic measurements of rate distribution [19–21]. The complexity comes from both the many possible ways of interbasin transitions of one conformational basin to another with slightly different initial or final conformational substates within each basin and intrabasin transitions of one conformational substate to another within each specific conformational basin. Our simulations and experiments all show approximately a bi-exponential fit for the correlation function with similar numerical ratios of one time scale with another between experiment and theory (Figures 2 and 3D). Thus, the theory and models provide the information of the inherent structure of the binding landscape. By probing kinetics using the single-molecule technique, one can explore the statistical nature and topography of the underlying binding energy landscape [19–21].

Figure 4 shows the free-energy profile *F*(*Q_f2_, Q_b_*) with different cooperativity (three-body force) parameters α. The basic global two-dimensional free-energy profiles do not change significantly (the relative positions of the free-energy minimum, maximum and saddle point, the free-energy barrier and the transition states). This means that the structures of the loosely bound and bound states observed in the experiment persist with different strengths of the underlying cooperative interactions. We also see that the free-energy barrier increases as the cooperativity α or the multibody force increases. The free energy is composed of both the contribution from energy and entropy. Bringing more residues together, although more stable energetically, is very costly in terms of entropy. Therefore, the overall effect is the increase of the free-energy barrier.

**Figure 4:**
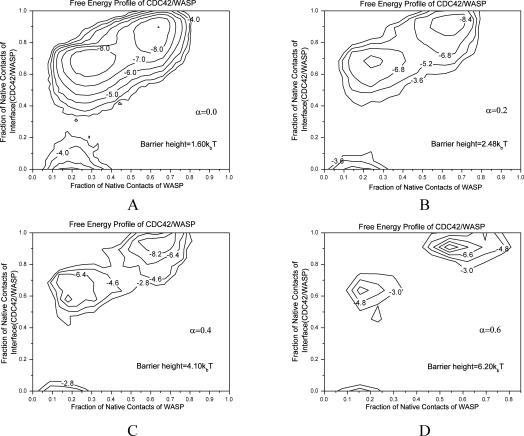
Two-Dimensional Free-Energy Landscape versus Interface Binding of Cdc42 and Folding CBD for Different α

### Characterization of Transition State Ensemble of the Cdc42-CBD Complex

On the basis of the landscape theory and Go model simulation, we can obtain free-energy profiles. We looked at the two-dimensional free-energy profiles in terms of binding and folding degrees of freedom of CBD with Cdc42. According to the definition of the transition state, we located it by finding the extremum between the minima of the free-energy profile. The extremum here is actually the saddle point of the free energy (first derivatives of the free energy are all equal to zero, and second derivatives of the free energy have negative and positive eigen values). The transition state is thus found to be at a particular position of the reaction coordinates (*Q_b_* = 0.7, *Q_f_*_2_ = 0.3). We have performed analysis on the φ values of Cdc42-CBD and its associated distribution to explore the nature of the binding transition state ensemble. For Cdc42-CBD, binding seems to proceed as a nucleation process. The distribution of φ values, which are shown in Figure 5A, shows prominent peaks near 1, indicating that there are certain residues with large φ values. These hot spots are crucial for the kinetic process and act as the nucleus or nucleation seeds of binding (see Figure 5B of the theoretical φ values along the sequence positions of CBD portions of WASP and Cdc42). The hot residues with high φ values are Phe37, Phe56 (Cdc42), Leu263, and Leu267 (CBD), respectively, along with others. These are quite consistent with the limited mutational experiments done so far [15–17]. The theoretical predictions on φ values can be used to guide further experiments in terms of which other hot residues to pick for study. Further mutational experiments on Cdc42-CBD are crucial in determining all the hot residues for flexible binding and uncovering the fundamental mechanisms for binding that accompany the large conformational transitions between disordered and ordered states.

**Figure 5:**
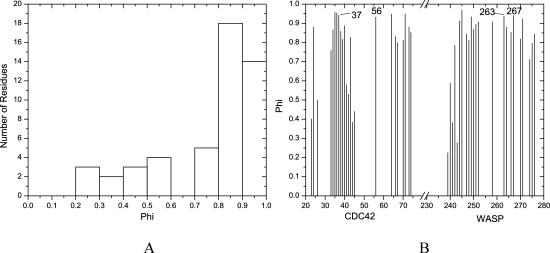
Distribution of Phi Values versus Protein-Primary Sequence (A) Distribution of phi values, and (B) phi values along the primary sequences of CBD portion of WASP and Cdc42.

Figure 6 shows the distribution of φ values and φ values versus the protein primary sequence with different cooperativity parameters (three-body force) α. The φ value of the four residues (#37, #56, #263, #267), which were also chosen to be studied in the experiments, increases as α increases. This means that as the underlying cooperative interactions become more important, these selected residues become more and more likely to cluster together, further confirming that they are among those key residues in the binding interface. Moreover, the distribution also shifts more toward φ = 1 as cooperativity becomes stronger, because cooperative interactions lead to the key residues being more distinct and important for the binding process. Thus, the process is more cooperative and nucleation-like, with the key residues acting as nucleation seeds. This resembles the first-order phase transitions in physics and chemistry (e.g., the water to vapor transition).

**Figure 6:**
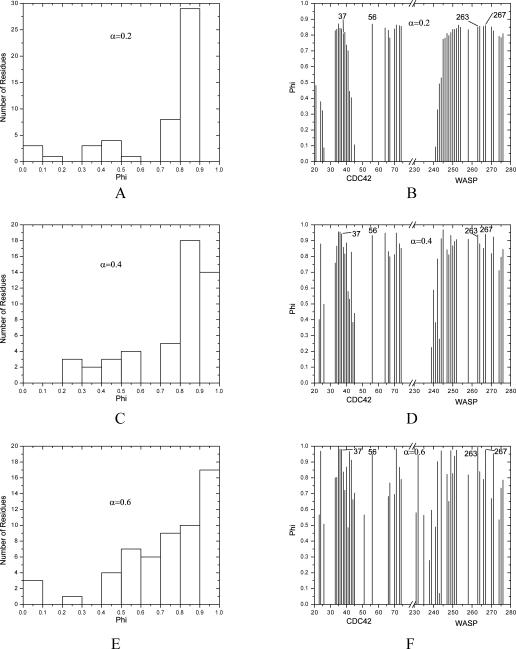
Distribution of Phi Values and Phi Values versus Protein-Primary Sequence for Different α

### Auto-Correlation Function of *Q_b_*

We also calculated the auto-correlation function c(Δ) of time interval Δ of the fraction of native interface contacts at different positions of *Q_b_* to characterize the time scales involved in the local binding dynamics:

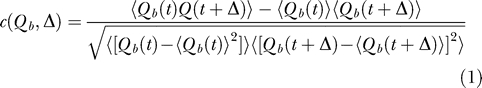
We see the correlation function decay with time Δ. We can fit the data using multiple exponentials and the overall time constant τ (from integration of the survival probability) for each *Q_b_* at simulation temperature *T_s_* in Figure 7.

**Figure 7:**
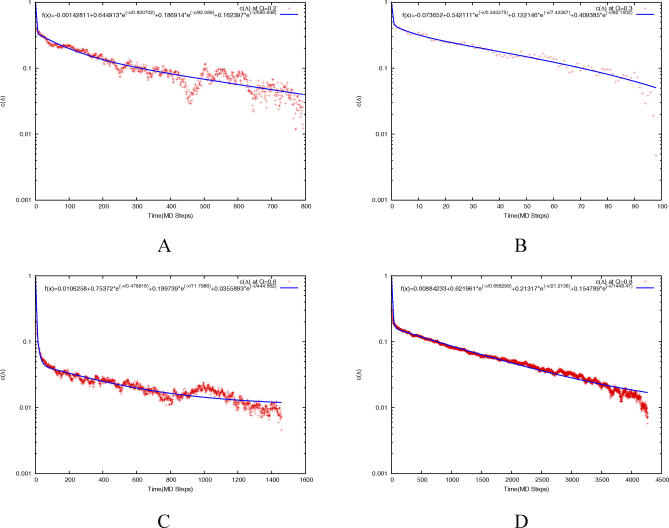
Auto-Correlations of Fraction of Interface Contacts *c*(Δ) at Different Positions of *Q_b_* and at *T* = *T_s_*

We see that in general the kinetics involve multi-exponential processes, which can typically be fitted with two or three exponentials. Since the kinetics is a probe to the underlying conformational energy landscape, the multi-exponential kinetics reflects the complexity or the inherent distribution of the energy landscape. The time decay and inherent complexity (multiple time-scale spread) in terms of the correlation function vary with the position *Q.* This maps out the local conformational energy landscape.

In Figure 8, we show the typical structures of the loosely bound, transition, and bound states, respectively, from our model simulations, along with their corresponding configuration and free-energy profiles, in terms of fraction of native (interface) contacts between Cdc42 and the CBD portion of WASP, as well as the fraction of native (folding) contacts of CBD itself. The loosely bound state has very limited spatial interface contacts. The transition state has accumulated significant interface contacts. The bound state has formed the most spatial interface contacts and reveals the most compact structure. There is a significant structural transition from the loosely bound to the bound state.

**Figure 8:**
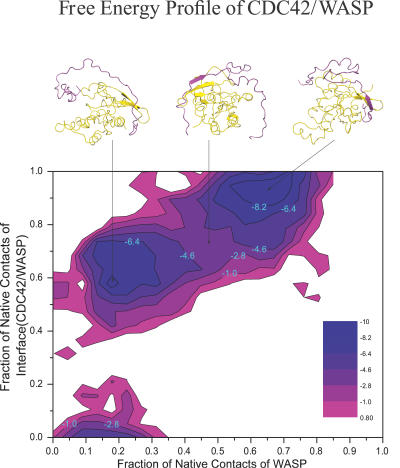
Typical Structures and their Corresponding Configuration in the Free-Energy Landscape Typical structures of loosely bound state (left structure), transition state (middle structure), and bound state (right structure) and their corresponding configuration in free-energy profiles in terms of fraction of native (interface) contacts between Cdc42 and CBD portion of WASP, as well as the fraction of native (folding) contacts of CBD itself.

## Materials and Methods

### Go model simulation.

In our theoretical investigations, based on native structure information and uniform stabilization binding energy, residue-level Go model simulations of Cdc42-protein/CBD binding dynamics were performed on a Pacific Northwest National Laboratory supercomputer. Fifty long-time trajectories of 20 million steps each were collected for a reliable statistical analysis. The native structure of the Cdc42-CBD binding complex had already been experimentally determined (PDB: 1cee) [15–18].

The Go model [27,28] takes into account only interactions that exist in the native structure, not energetic frustration, but includes only topological frustration or structural heterogeneity. We use here an off-lattice Go model, where each residue is represented by a single bead centered on its α-carbon (*C*_α_) position. Adjacent beads are pieced together into a polymer chain by means of a potential encoding bond length and angle constraints. The secondary structure is encoded in the dihedral angle potential and the nonbonded native contact potential. The interaction energy *U* at a given protein conformation Γ is given by:

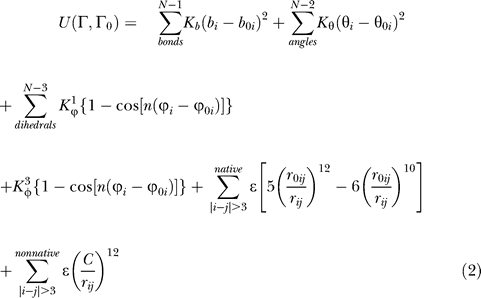
The first three terms in this equation represent the energies from backbone chemical bond vibrations and dihedral rotations. The fourth term represents the native interaction energy contribution to binding between two residues *i* and *j*, and the fifth term represents the nonnative interaction energy contribution to binding between two residues. In the equation, *b_i_*, θ*_i_*, and φ*_i_* stand for the *i*th virtual bond length between *i*th and (*i* + 1)th residue, the virtual bond angle between (*i* – 1)th and *i*th bonds, and the virtual dihedral angle around the *i*th bond in conformation Γ, respectively. The parameters *b*_0*i*_, θ_0*i*_, and φ_0*i*_ stand for the corresponding variables at the native structure Γ_0_. These three terms control the local conformations within four residues. In the framework of the model, all native contacts in the fourth term are represented by the 10–12 Lennard Jones potential form without any discrimination between the various chemical types of interaction [27,28]. The *r_ij_* and *r*_0*ij*_ are the *C*_α_ − *C*_α_ distances between the contacting residues *i* and *j* in conformation Γ and Γ_0_ (the PDB structure), respectively. In the summation over nonnative contacts in the fifth term, *C* (= 4.0Å) parameterizes the excluded volume repulsion between residue pairs that do not belong to the given native contact set. The last two terms control the nonlocal interactions. The first of the last two terms as mentioned gives the interactions among residues that are close in native structure. The last term is repulsive for nonnative interactions, so that this type of interaction is not preferred. In this paper, all temperatures and energies are reported in units of 4.0Å. For other parameters, we use similar values that have been used in several folding studies [4–6,27,28], namely, *K_b_* = 100.0, *K*_θ_ = 20.0, 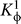 = 1.0, 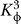 = 0.5, and ɛ = 1.0.

To enhance the sampling of binding events, we tried a method of linking the two monomers of the dimer by the center of mass constraint to a distance. The center of mass constraint acts to hold the two unbound subunits (folded or unfolded) in a close proximity during their motions, essentially enhancing the local concentrations. The center of mass constraint distance was determined approximately by the distance between the C terminus of subunit A and the N terminus of subunit B. This length was sufficient to ensure that the center of mass constraint would not interfere with any intra- or intersubunit contacts that stabilized the folded dimer. To optimize its conformation with respect to the dimer, a minimization was performed on the center of mass constraint, including the two residues to which the center of mass constraint is directly connected. For the studied dimers, multiple constant-temperature MD simulations were performed (using the simulation package AMBER6 as an integrator) starting from either the dimeric conformation or the unfolded monomers.

We noted that the time scale involved in the simulations was an “effective” time scale. Because of the funneled-landscape coarse-grained energy function at the residue level and the bias toward the native structure, the computational difficulty of reaching the native binding state in finite time was overcome. In other words, the kinetic simulation times of reaching the native state are significantly shortened on a funneled energy landscape. So, within the normal MD simulation “effective” times, the whole dynamic process, from nonnative to native binding states, can be followed and studied.

We have performed Go model MD simulations at different temperatures using the replica exchange method to sufficiently explore the conformation space [42–45]. In this way, we can use the weighted histogram method to obtain the free-energy profiles [42,46,47]. Once the free-energy profiles are determined, we can calculate the heat capacity as a function of temperature. We define *T_f_* as the temperature at which the heat capacity is at its peak. This is the transition temperature from nonnative states to the native state. From the free-energy landscape profiles, we found loosely bound states and bound states, which, as were observed from the single-molecule experiments [19–21], coexist at certain temperature ranges below *T_f_*. We chose the simulation temperature *T_s_* so that the free energy at the loosely bound and bound states was about equal. In this way, the probability of finding loosely bound states and bound states was about equal, corresponding to the observed kinetic two-state behavior in single-molecule experiments [19–21]. We believe this temperature in the simulation is close to the one in the actual experiments. We find that *T_s_* = 0.944*T_f_* (Figure 9). Here, *T_f_* is determined by the peak of the heat capacity *C_v_*(*T)* with respect to temperature, which resembles the phase transition temperature from a completely nonnative to a native binding state. Note that *T_f_* = 485 in our simulation. The value of *T_f_* is not the absolute temperature in degrees Kelvin. It is in the simulation unit. Here, we scaled this temperature to be 1 for simplicity:


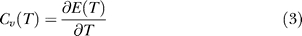


**Figure 9:**
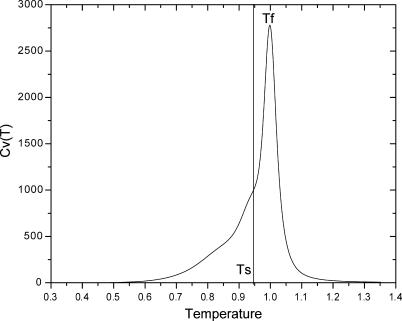
Heat Capacity *C_v_*(*T*) The simulation temperature *T_s_* = 0.944, and melting temperature *T_f_* = 1.0. The *T_f_* is defined from the peak of *C_v_*, and *T_s_* is determined from two-dimensional free-energy profile *F*(*Q_f_*_2_,*Q_b_*)**.** The simulation temperature was chosen so that the free-energy values at the loosely bound and bound states are equal.

### Three-body interactions inferred free energy and φ value calculations.

The microscopic interactions at the atomic level are all two-body. However, here we used a coarse-grained description at the residue level and also integrated out the water molecules, which interact with the residues. So, the effective interactions among residues in this coarse-grained description are expected to be multibody. Three-body interactions can improve the accuracy of the free-energy profile as well as the kinetics and f value analysis. Our three-body interaction scheme was based on previous work [39,40,41]. If three *C*_α_ form native contacts with each other within a given cutoff distance (for example, 4.7Å), they are counted as one three-body interaction. For a given conformation, the total energy of two-body interactions is *E*_2_ = ɛ*QN*_2_ and of three-body interactions is *E*_3_ = ɛ_3_ *Q*_3_ *N*_3_. The Lennard-Jones-like potential depth in this expression is given as ɛ = 1.0 and ɛ_3_ = ɛ*N*_2_/*N*_3_, and *Q* and *Q*_3_ are the fractions of native contact pairs and triplet contacts present in that conformation. *N*_2_ and *N*_3_ are the total numbers of native two-body and three-body contacts, respectively. If a parameter α (0 ≤ α ≤ 1) is used to control the relative contribution from two-body pair and three-body triple contacts, the native energy becomes

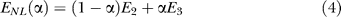
and the new free energy is given as

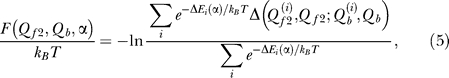
where the sum is of all sampled conformations *i*, and 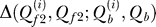 is a delta function that selects only those states where 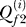 = *Q_f_*_2_, 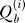 = *Q_b_* and Δ*E*(α) = *E_NL_*(α) − *E*_2_.

Studying the transition state properties, especially the inhomogeneous distribution of contacts between residues, will help understand the mechanism of binding by locating the sites of the nucleation for binding. Thus, the free-energy landscape is not uniform in space, and the local parts of the landscape can be perturbed by mutations. This causes changes in both equilibrium constant and kinetics of binding. The ratio φ*_i_* is defined as the change of kinetic rate and equilibrium constant upon mutation at residue *i* [48]:

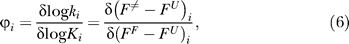
where δlog*k_i_* is the change of the logarithmic kinetic rate for binding upon mutation of residue *i*, and δlog*K_i_* is the change of the logarithmic equilibrium constant upon mutation of residue *i*. *F* ^≠^, *F^U^*, and *F^F^* represent transition-state free energy, unbound-state free energy, and native-bound free energy upon mutation change of residue *i*, respectively. If φ*_i_* = 1, then it means the free-energy change of the transition state is equal to the free-energy change of the native state upon the mutation of the particular residue *i*. This implies that this particular residue is crucial for binding. On the other hand, if φ*_i_* = 0, then it means the free-energy change of the transition state is equal to the free-energy change of the nonnative state upon the mutation of the particular residue *i*. The value of φ, therefore, provides an important characterization for particular residues at the transition state ensemble. Therefore, φ has a crude statistical mechanical interpretation of spectroscopy (characterization) or marker of the transition state ensemble [48]. In experiments, some “hot residues” with high Φ values in transition clustered together. This indicates that the nucleus for binding can be identified.

In our model, φ can be approximately calculated through the following equation:

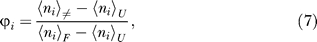
where 〈*n_i_*〉 is the thermal mean value of the number of two-body interaction contacts for residue *i* over all the corresponding states, and the ≠, *U*, and *F* subscripts represent the transition state, unbound state, and native bound state, respectively. These three states are determined from the free-energy profile mentioned earlier.

In the presence of three-body interactions, the φ values can be approximately calculated as

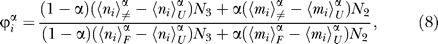
where *m_i_* is the number of three-body interactions in which residue *i* is involved, and the superscript α refers to averaging the three states in the presence of three-body energy, giving the relative importance of the three-body interactions. When α → 0, Equation 8 reduces to Equation 7.

### Summary.

In this work, we studied biomolecular recognition dynamics using single-molecule experiments and coarse-grained MD simulations. Our model provides a quantitative characterization of the flexible binding free-energy landscape from which we can explore fundamental mechanisms and the roles of flexibility in binding. We find that single-molecule spectroscopy is a powerful approach in studying the fundamental mechanism of flexible binding in protein–protein recognitions. The complex nature of the conformational fluctuations upon binding found in the single-molecule experiments directly reflects the topography of the binding landscape and reveals the cooperative nature of binding-folding coupling (with its large conformational changes), which results in loosely bound and bound states [19–21].

Our results show that some proteins require binding with others in order to be structured, implying that binding and folding (or large conformational changes) are intimately coupled. In our system of Cdc42-CBD, the extreme, significant binding occurs first before the subsequent cooperative binding-folding coupling reaches the native binding complex. This finding is in contrast to the conventional picture of a mechanism in which binding proceeds with complete folding first and then further binding reaches the native binding complex [4–6].

Limited mutational experiments [15–17] show some hot spots that are crucial for flexible binding. The theoretical approach can help identify these hot residues for engineering design and experiments that will further unravel the fundamental mechanisms of binding. Conversely, experiments can provide a test ground for the construction of theories on the binding energy landscape.

Our findings of large-amplitude conformational fluctuations in the interactions of protein complexes are consistent with recent nuclear magnetic resonance static and ensemble-averaged structural analysis [15–17]. It is reasonable to suggest that structural transitions of flexible conformational domains are probably common in biomolecular recognition processes.

It is worthwhile to note that there are advantages to biomolecular recognition with flexibility, which can help to find a best-fit by adjusting conformations [3,49,50] and can result in enhanced speed of recognition through a larger capture radius for specific binding sites [4–6].

It is interesting that the CBD portion of the WASP binding with other proteins is common to various cellular and signal transduction processes in different species [15–18]. For example, CBD binds proteins closely related to Cdc42 from other members of the Rho family. It responds to both Cdc42 and the closely related protein TC10, which bind WASP with a similar affinity [18]. So, the flexible binding mechanism illustrated here might be quite general for many similar biological processes.

The two-dimensional nature of the cooperative binding-folding from loosely bound and bound states suggests that in order to characterize this process, one needs simultaneously to monitor the binding degrees of freedom as well as the conformational or folding degrees of freedom (Figure 3A). The conformational or folding and the interface degrees of freedom have been studied separately through single-molecule and bulk measurements as well as through nuclear magnetic resonance techniques [7–17]. It would be ideal to monitor the dynamic binding process and at the same time to keep track of the associated folding or large conformational changes in bulk and single-molecule measurements. This is a challenge for future experiments.

## Abbreviations

GTP: guanosine 5-triphosphate
MD: molecular dynamics
WASP: Wiskott-Aldrich syndrome protein
